# Feasibility of a pretrial diversion pilot in Kent County, Rhode Island

**DOI:** 10.1186/1940-0640-10-S1-A68

**Published:** 2015-02-20

**Authors:** Anna F Tomasulo, Nickolas Zaller, Bradley W Brockmann, Jennifer Lerch, Scott T Walters, Faye S Taxman

**Affiliations:** 1 Center for Prisoner Health and Human Rights, The Miriam Hospital, Providence, RI, 02906, USA; 2 Department of Criminology, Law & Society, Center for Advancing Correctional Excellence, George Mason University, Fairfax, VA, 22030, USA; 3 Department of Behavioral and Community Health, University of North Texas Health Science Center, Fort Worth, TX, 76107, USA

## Background

The U.S. correctional population is disproportionately burdened with chronic health conditions, particularly mental health and substance use disorders. Though inmates receive health care, linkages to care upon release are often poor or nonexistent. Further, the magnitude of the justice-involved population strains state and Federal finances. The annual cost per offender awaiting trial in Rhode Island is approximately $40,000. A diversion program (an alternative sentence diverting from incarceration and offering potential relief to courts and better outcomes for offenders) with linkages to health care has the potential to reduce costs by addressing the underlying physical, mental, and behavioral health issues keeping individuals in the justice system.

We describe a diversion pilot program and our observations thus far. In partnership with Brown University and The Center for Prisoner Health and Human Rights, Justice Assistance, a nonprofit organization in Cranston, Rhode Island, received support from the Rhode Island General Assembly to develop a program to reduce the Rhode Island awaiting population by linking participants to community-based health care. The pilot program developed, Office of Community Alternatives (OCA), tests an intensive case management model. MAPIT (Motivational Assessment Program to Initiate Treatment) is an Internet-based motivational interviewing program piloted as a component of the OCA program.

## Objective and measures

To determine OCA feasibility at the superior court level, we explore: whether clients engage in treatment; levels of support from courts, the offices of the public defender and attorney general; whether clients return to prison; how much time case managers spend with clients; and client satisfaction with MAPIT.

## Observations

As of October 1, 2014, 25 individuals were enrolled in the OCA. Twenty-five clients completed a substance use assessment; 13 completed a mental health assessment; 10 completed a physical health assessment; 24 were prescribed treatment, and 24 began treatment. Nine individuals were terminated from the OCA due to noncompliance. Eight of those terminated returned to the adult correctional institute with new charges, while one is out with a bench warrant. Three clients completed the OCA program. None of those who have completed the program have recidivated.

Given the unprecedented collaborations in support of this program, there has been a high level of scrutiny over the referral process. This has significantly slowed enrollment. Recent discussions make us hopeful that in the future we can establish strict eligibility criteria.

## Next steps

We hope to conduct qualitative interviews with clients and stakeholders, and begin evaluating impacts of OCA and MAPIT.

